# Effect of Barley on Postprandial Blood Glucose Response and Appetite in Healthy Individuals: A Randomized, Double-Blind, Placebo-Controlled Trial

**DOI:** 10.3390/nu16223899

**Published:** 2024-11-15

**Authors:** In-Sook Kim, Soo-yeon Park, Min Ju Park, Kyeong Jin Kim, Ji Yeon Kim

**Affiliations:** 1 Department of Nano Bio Engineering, Seoul National University of Science and Technology, 232, Gongneung-ro, Nowon-gu, Seoul 01811, Republic of Korea; 2 Department of Food Science and Biotechnology, Seoul National University of Science and Technology, 232, Gongneung-ro, Nowon-gu, Seoul 01811, Republic of Korea

**Keywords:** barley, dietary fiber, β-glucan, postprandial blood glucose, diabetes, appetite

## Abstract

**Background/Objectives:** Barley dietary fiber (BDF), particularly β-glucan, has shown potential in modulating postprandial glycemic responses and improving metabolic health. This study aimed to assess the effects of Saechalssalbori (*Hordeum vulgare* L.), a glutinous barley variety rich in β-glucan, on postprandial blood glucose, insulin, glucagon, triglycerides, and appetite-related hormones in healthy adults. **Methods:** In this randomized, double-blind, placebo-controlled, crossover trial, healthy adults (*n* = 67) with fasting blood glucose levels below 126 mg/dL were assigned to consume either BDF or placebo (rice flour). Fasting and postprandial blood samples were collected at 30, 60, 120, and 180 min after consumption. Blood glucose, insulin, glucagon, triglycerides, and appetite-related hormones (ghrelin, PYY) were measured, and appetite was assessed using the visual analog scale (VAS). The study was approved by the Institutional Review Board (CHAMC 2022-08-040-007) and registered (KCT0009166). **Results:** BDF consumption significantly delayed the postprandial increase in blood glucose compared with placebo, reduced insulin secretion, and slightly increased glucagon and triglycerides. BDF also lowered hunger and increased satiety, with associated increases in ghrelin and PYY levels. **Conclusions:** BDF consumption, particularly from β-glucan-rich barley, may improve postprandial glycemic control and suppress appetite, making it a promising dietary intervention for managing metabolic conditions such as diabetes. Further studies are needed to explore its long-term impact on glycemic variability.

## 1. Introduction

Diabetes is a chronic metabolic disorder characterized by hyperglycemia, caused by either abnormal insulin secretion or insulin resistance, and represents a significant and growing global public health concern [1]. In 2022, the International Diabetes Federation predicted that by 2045, three-quarters of all diabetes cases will occur in low- and middle-income countries [2]. Furthermore, using the most recent data and methodologies from the Global Burden of Diseases, Injuries, and Risk Factors Study (1990–2021), it is predicted that the global number of diabetes cases will exceed 1.3 billion by 2050, more than doubling from today [3]. According to the 2022 National Health and Nutrition Examination Survey in Korea, the prevalence of diabetes in adults aged > 19 years was 11.2% in men and 6.9% in women, with an overall prevalence of 9.1%. Notably, diabetes prevalence was higher in individuals from lower-income groups (11.4%) compared with those from higher-income groups (7.6%); however, dietary fiber intake was slightly higher in the higher-income groups (22.7%) than in lower-income groups (21%) [4]. Diabetes is mainly classified into types 1 and 2. Type 1 diabetes is a chronic autoimmune disease mainly caused by genetic predisposition and autoimmune destruction of pancreatic β cells [5,6]. Type 2 diabetes (T2D), which accounts for over 90% of diabetes cases, is characterized by insulin resistance and impaired insulin secretion, leading to dysregulated glucose and lipid metabolism [7]. T2D is often considered a lifestyle-related disease, heavily influenced by factors such as excess carbohydrate intake, physical inactivity, and obesity [8,9,10].

In this regard, dietary management plays a crucial role in T2D prevention and management, particularly through the intake of dietary fiber. Studies have shown that barley-derived β-glucan has the potential to improve various health markers, including diabetes, obesity, and glucose regulation, in both healthy individuals and those with metabolic disorders [11,12,13,14]. Over recent decades, extensive scientific evidence has demonstrated the health benefits of β-glucans from barley and oats in lowering cholesterol and regulating blood glucose, leading both the U.S. Food and Drug Administration (FDA) and the European Food Safety Authority (EFSA) to approve health claims for their role in reducing cardiovascular disease and controlling postprandial glycemic responses [15,16,17,18]. Soluble dietary fibers such as β-glucan, pectin, and hemicelluloses have a high water-holding capacity, providing satiety in the stomach and improving glucose tolerance in diabetic patients [19]. β-glucan is particularly abundant in the cell walls of the endosperm of barley and oats, making up around 70%, and is also present in the aleurone layer [20]. Barley β-glucan consists of mixed-linkage β-D-glucan with β-1,3 and β-1,4 glucoside bonds in a 3:7 ratio with a content of 3.0–6.9%, of which 38–69% is water soluble [21]. These structural characteristics are crucial to β-glucan’s water solubility, viscosity, and gelation properties, with interactions between other compounds serving as key factors in determining the functionality of barley β-glucan in food systems and its influence on postprandial responses [22].

Saechalssalbori (*Hordeum vulgare* L.), developed by the Rural Development Administration in 1995, is a glutinous naked barley variety. This variety features well-separated husks, making it suitable for consumption, and is characterized by large grains, good cooking quality, early harvest, and strong resistance to environmental stress. Compared with traditional glutinous barley varieties, Saechalssalbori has a higher starch content, a whiter appearance, and an increased β-glucan content. The glutinous starch structure of Saechalssalbori exhibits significantly higher water absorption and spreadability compared with non-glutinous barley, facilitating easier gelatinization during cooking [23]. Its high viscosity is also expected to form a strong gel, which may positively impact digestion, absorption, and metabolism in the intestines. However, there is currently a lack of research on the postprandial blood glucose effects of Saechalssalbori. Therefore, this study aims to investigate the effect of consuming dietary fiber powder from Saechalssalbori produced using a standardized manufacturing process to consistently regulate the fiber content, including β-glucan, on improving postprandial blood glucose levels.

## 2. Materials and Methods

### 2.1. Study Materials

The barley used in this study was Saechalssalbori (*Hordeum vulgare* L.), a certified seed variety supplied by the Rural Development Administration in 2022. The barley was harvested in June 2023 in Pyeongtaek, Gyeonggi Province, South Korea, and immediately hot-air dried. It was milled to a 7% polish rate, maintaining approximately 95% of the grain’s bran, endosperm, and aleurone layers. The processing and sterilization of the barley were conducted at the Korea Food Industry Cluster Promotion Agency (Iksan, Republic of Korea), after which the barley was powdered. In the final formulation, 88.7% of the total composition consisted of barley powder. Additional ingredients included enzyme-treated stevia (1.5%), roasted rice flavor powder (7.3%), and roasted rice fragrance powder (2.5%). For the placebo product, 88.7% rice flour was used in place of barley powder, ensuring consistency in the composition between the two products except for the active ingredient. This powder formulation allowed for precise dosing in the study, with each bottle containing 150 g of product, of which 7 g consisted of soluble barley fiber in the test product.

Subjects consumed the test and placebo products by consuming one bottle per serving. Each bottle was provided in a shaker to facilitate mixing, with participants instructed to add 500 mL of water to fully dissolve the powder, creating a beverage for easy consumption. This standardized preparation method ensured consistency in the volume and concentration of each serving across all participants.

Each 150 g serving of the barley dietary fiber product contained a total of 576.89 kcal, with a macronutrient composition of 73.5% energy from carbohydrates (106.0 g), 18.0% from protein (26.0 g), and 8.5% from fat (5.4 g). The carbohydrate content included complex carbohydrates and dietary fiber (24.7 g), with 2.6 g of sugars (including fructose, glucose, sucrose, lactose, maltose, and galactose). In contrast, the placebo product (rice flour) provided a total of 539.36 kcal per 150 g serving. Its macronutrient profile consisted of 90.2% energy from carbohydrates (121.6 g), 7.5% from protein (10.1 g), and 2.3% from fat (1.4 g). The carbohydrates in the placebo product included 0.4 g of fiber and 6.2 g of sugars.

### 2.2. Study Design

This study followed a randomized, double-blind, placebo-controlled, crossover design. Participants were randomly assigned to either the placebo group or the treatment (BDF) group in a 1:1 ratio one day prior to Visit 2 (Week 1) using a randomization method. At Visit 3 (Week 2), participants were crossed over to the alternate group. The study began with Visit 1 (Week 0), during which participants voluntarily signed informed consent forms. Eligibility was determined based on inclusion and exclusion criteria. After a 1-week run-in period, eligible participants were randomly assigned to either the BDF or placebo group according to the order of registration at Visit 2 (Week 1). They consumed either the treatment product or the placebo product, followed by a 1-week washout period. At Visit 3 (Week 2), participants were crossed over to the other group and consumed the respective product. The study was conducted at CHA Bundang Medical Center, CHA University (Seongnam, Republic of Korea). The study protocols were approved by the Institutional Review Board of CHA Bundang Medical Center, CHA University (CHAMC 2022-08-040-007). The study was registered with the WHO International Clinical Trials Registry Platform under the identification number KCT0009166.

### 2.3. Participants

Participants in the study were healthy male and female adults aged between 20 and 65 years with fasting blood glucose levels below 126 mg/dL, recruited through poster advertisement on a website. Individuals were excluded if they met any of the following conditions: continuous use of functional foods, herbal medicines, or prescription medicines within 4 weeks prior to the first visit; a body mass index (BMI) of ≤18.5 kg/m^2^; clinically significant symptoms or active diseases; a high-quality diet (recommended food score > 36 points); regular intense exercise (≥10 h/week); weight change exceeding 4 kg or adherence to a weight control diet within 4 weeks of visit 1; alcohol addiction; excessive smoking (≥20 cigarettes/day); difficulty using e-diary (those who have difficulty using smartphones or computers); participation in other clinical studies within 4 weeks of the first visit; use of research drugs or functional foods (except if they had not taken the drugs or functional foods); pregnancy or lactation; plans for pregnancy; hypersensitivity to the ingredients contained in the treatment and placebo products; history of severe food allergy reactions; or if they were deemed inappropriate by the researcher to participate in the study. Participants were interviewed individually to obtain background information, including age, anthropometric exercise, alcohol consumption, smoking, and medical history, and the protocol was fully explained to the participants at the screening visit. All participants provided written informed consent prior to enrollment.

### 2.4. Sample Size Calculation

The sample size for this crossover study was calculated to ensure adequate power to detect differences in postprandial blood glucose-related indicators between the placebo and treatment groups. Based on previous studies examining the effects of dietary interventions on blood glucose and insulin responses, we estimated a required sample size of 28 participants per sequence. Studies by Bays et al. [24], Higa et al. [12], Ames et al. [25], and Kim et al. [26] provided reference values for inter-group differences in glucose and insulin levels at various time points. Using these data, along with a significance level (α) of 5% (two-tailed) and a power of 80% (β = 0.2), the sample size was calculated. To account for an expected dropout rate of 20%, a total of 70 participants were recruited, with 35 participants assigned to each sequence.

### 2.5. Protocol

On the first day of the study, participants were assigned a randomization number provided the previous day and were assessed for sex, date of birth, age, and BMI. Their usual exercise habits were also recorded, and for female participants, additional information regarding menstruation and the duration of amenorrhea was collected. At Visit 2 (Week 1), a family history of diabetes was assessed, and both Visit 2 (Week 1) and Visit 3 (Week 2) included evaluations using the Global Physical Activity Questionnaire (GPAQ) and the Recommended Food Score (RFS).

All participants arrived after fasting for at least 12 h to measure fasting blood glucose. For women, a urine HCG pregnancy test was conducted, except for those confirmed as postmenopausal (amenorrhea for over 24 months). Participants received education on dietary and lifestyle guidelines, as well as instructions on how to maintain an e-diary. Compliance with these guidelines was reviewed at both Visit 2 (Week 1) and Visit 3 (Week 2), and participants deemed suitable for further evaluation completed additional blood and urine tests after fasting for 12 h at Visit 2 (Week 1).

After one week, at Visit 3 (Week 2), participants again arrived after fasting for 12 h for analysis of fasting blood glucose, insulin, glucagon, triglycerides, and appetite using a visual analog scale (VAS). After consuming either the BDF or placebo product with 30 g of glucose loading, blood samples were taken at 30, 60, 120, and 180 min to analyze. Key indices such as postprandial glucose, insulin levels, area under the curve (AUC) for glucose and insulin, and the insulin/glucose ratio were calculated, along with additional biochemical markers for further analysis.

### 2.6. Visual Analog Scale (VAS)

A visual analog scale (VAS) was used to assess participants’ subjective appetite sensations, including hunger, satiety, and prospective food intake. VAS assessments were conducted at baseline (0 min), following an overnight fast of at least 12 h, and subsequently at 30, 60, 120, and 180 min after the ingestion of either the BDF or placebo product with a 30 g glucose load.

The VAS consisted of a 100 mm horizontal line anchored with “not at all” at 0 mm and “extremely” at 100 mm. Participants were instructed to mark a point along the line that best represented their current sensation at each specified time point. To ensure consistency, all participants received standardized instructions on completing the VAS. This protocol allowed for the systematic evaluation of appetite responses over the postprandial period, facilitating comparisons between time points and treatment conditions.

### 2.7. Ghrelin and PYY Enzyme-Linked Immunosorbent Assays (ELISAs)

Plasma was separated by centrifugation at 1500× *g* for 10 min at 4 °C, aliquoted, and stored at −80 °C until analysis. Plasma concentrations of the appetite-regulating hormones ghrelin and peptide YY (PYY) were quantified using ELISA kits (Human Ghrelin ELISA Kit and Human PYY ELISA Kit, Invitrogen, Thermo Fisher Scientific, Inc., Waltham, MA, USA).

For the ghrelin assay, 100 μL of each plasma sample or standard solution was added to individual wells, followed by 50 μL of biotin conjugate. The plate was incubated at room temperature for 2 h. After washing, 100 μL of Streptavidin-HRP was added and incubated for 1 h. For the PYY assay, 100 μL of each plasma sample or standard solution was added to the wells, and the plate was incubated for 2.5 h with shaking. After this incubation, 100 μL of biotin conjugate was added and incubated for an additional hour. Following washing, 100 μL of Streptavidin-HRP was added and incubated for 45 min. In both assays, after the final washing step, 100 μL of TMB substrate was added and incubated for 30 min. The reaction was stopped by adding 50 μL of stop solution, and absorbance was measured at 450 nm using a microplate reader (BioTek Instruments, Inc., Winooski, VT, USA).

### 2.8. Statistical Analysis

The results are presented as the means ± standard errors (SEs). All statistical analyses were performed using SAS 9.4 (SAS Institute, Cary, NC, USA). Postprandial blood glucose, insulin, glucagon, triglyceride, appetite VAS, and appetite hormones were estimated with a linear mixed-effects model (LMM) to the analysis of differences between groups over multiple time points considering group, sequence, visit, and time as fixed effect and subject within the sequence as a random effect. The area under the curve (AUC) was calculated using the trapezoidal rule. The maximum concentration (C_max_) at which a postprandial response was observed and the time point at which the maximum concentration (T_max_) was achieved were calculated as summary values.

Alcohol and smoke amount, physical activity, and vital signs were compared using LMM between the groups. In adverse events, Fisher’s exact test was used to compare the differences in the number of subjects between the groups.

## 3. Results

### 3.1. Subject Characteristics

**Figure 1:**
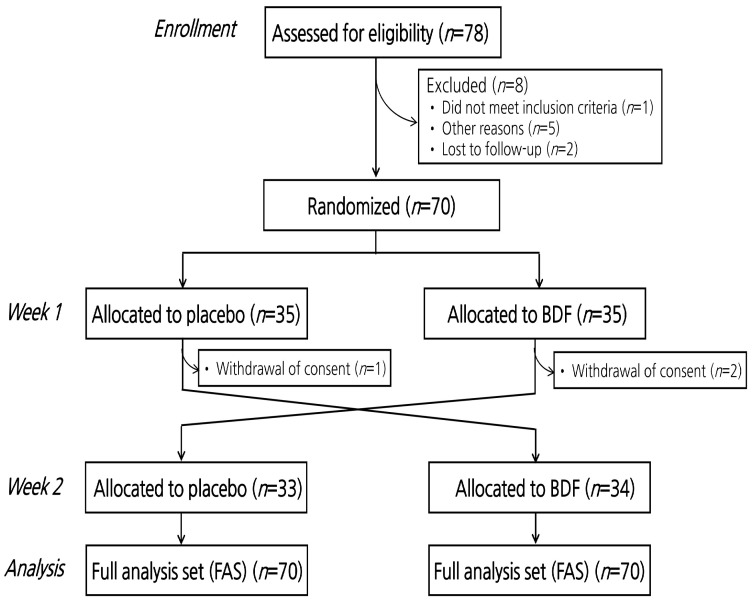
CONSORT diagram for flow of subjects through the study. BDF, barley dietary fiber. Of the total of 78 applicants, 67 were included in the study after excluding eight individuals who did not meet the selection criteria and three who voluntarily withdrew after the second interview. These participants were then randomly assigned to two groups for clinical trials and functional evaluations conducted through a crossover design.

A CONSORT diagram for the flow of subjects in the study is shown in Figure 1. A total of 78 individuals were screened, and 70 participants were selected for the study. These participants were randomly assigned into two groups: 35 in the placebo group and 35 in the BDF group, all of whom completed Visit 2 (Week 1). Subsequently, 3 participants withdrew consent, resulting in 33 participants in the placebo group and 34 in the BDF group completing Visit 3 (Week 2). The baseline characteristics of the participants are presented in Table 1. The average age of the participants was 35.3 ± 1.1 years, and their mean fasting blood glucose level was 95.7 ± 0.9 mg/dL.

### 3.2. Effects of BDF on Postprandial Blood Glucose and Insulin Secretion Levels

**Figure 2:**
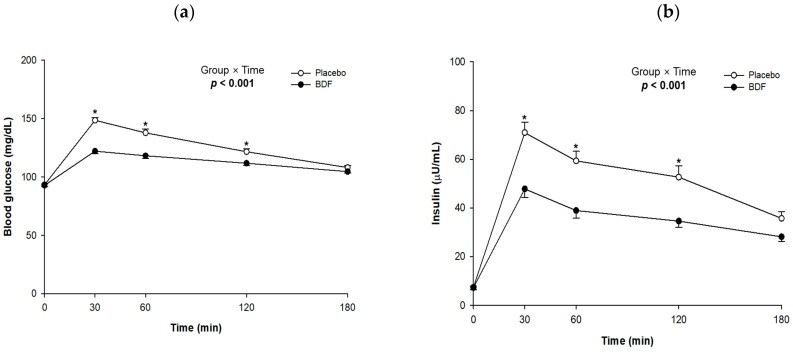
Effects of BDF on postprandial (**a**) blood glucose and (**b**) insulin secretion levels. BDF, barley dietary fiber. Each line represents mean ± SE. Linear mixed-effect model was used to analyze the difference between the groups and the effects of group × time. * *p* value < 0.05.

The analysis of both postprandial blood glucose and insulin levels is presented in Figure 2 and Table 2. Compared with the placebo group, the BDF group showed significant reductions in postprandial blood glucose at 30 min (β = −26.0, *p* < 0.001), 60 min (β = −19.2, *p* < 0.001), and 120 min (β = −9.4, *p* = 0.004), with a significant interaction effect over the 180 min period (*p* < 0.001). Similarly, postprandial insulin levels were significantly lower in the test group at 30 min (β = −23.0, *p* < 0.001), 60 min (β = −20.3, *p* < 0.001), and 120 min (β = −18.0, *p* < 0.001), with a significant interaction effect over the 180 min period (*p* < 0.001).

The area under the curve (AUC) for both blood glucose and insulin significantly decreased at all time intervals. For blood glucose, 0–30 min (β = −398, *p* < 0.001), 30–60 min (β = −684, *p* < 0.001), 60–120 min (β = −869, *p* < 0.001), and 120–180 min (β = −387, *p* = 0.001); for insulin, 0–30 min (β = −355, *p* < 0.001), 30–60 min (β = −670, *p* < 0.001), 60–120 min (β = −1186, *p* < 0.001), and 120–180 min (β = −787, *p* < 0.001). Cumulative AUC values for both blood glucose and insulin also showed significant reductions across all intervals.

Additionally, the maximum blood glucose concentration (C_max_) was significantly reduced (β = −24.7, *p* < 0.001), along with the maximum insulin concentration (C_max_) (β = −28.3, *p* < 0.001). However, while the time to reach maximum glucose concentration (T_max_) was significantly increased (β = 17.0, *p* = 0.002), there was no significant change in T_max_ for insulin levels.

### 3.3. Effects of BDF on Postprandial Blood Glucagon and Triglycerides Concentration

**Figure 3:**
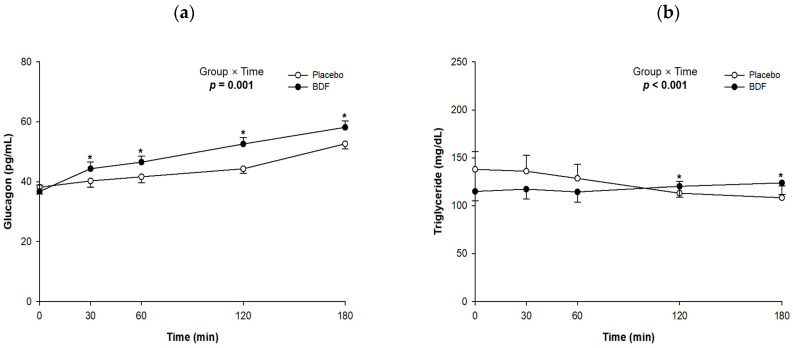
Effects of BDF on postprandial (**a**) glucagon and (**b**) triglyceride levels. BDF, barley dietary fiber. Each line represents mean ± SE. Linear mixed-effect model was used to analyze the difference between the groups and the effects of group × time. * *p* value < 0.05.

Effects of BDF on postprandial blood glucagon and triglycerides levels shown in Figure 3. Glucagon concentrations were similar before consumption, measuring 38.2 ± 2.3 pg/mL in the placebo group and 36.8 ± 2.1 pg/mL in the BDF group. However, after 120 min, glucagon levels increased to 44.3 ± 1.5 pg/mL in the placebo group and 52.6 ± 2.2 pg/mL in the BDF group. A significant interaction effect was observed over the 180 min period (*p* = 0.001).

In terms of blood triglyceride levels, contrasting results were noted between the placebo and BDF groups. The placebo group exhibited a gradual decrease in triglyceride levels from 138.0 ± 18.5 mg/dL immediately after consumption to 108.3 ± 12.2 mg/dL after 180 min. In contrast, the BDF group showed an increase from 115.0 ± 9.7 to 123.9 ± 12.3 mg/dL over the same period. Although a significant interaction effect was observed over the 180 min period (*p* < 0.001), the changes remained within the normal range.

### 3.4. Effects of BDF on Hunger, Satiety, and Appetite Hormone (Ghrelin and PYY) Levels

**Figure 4:**
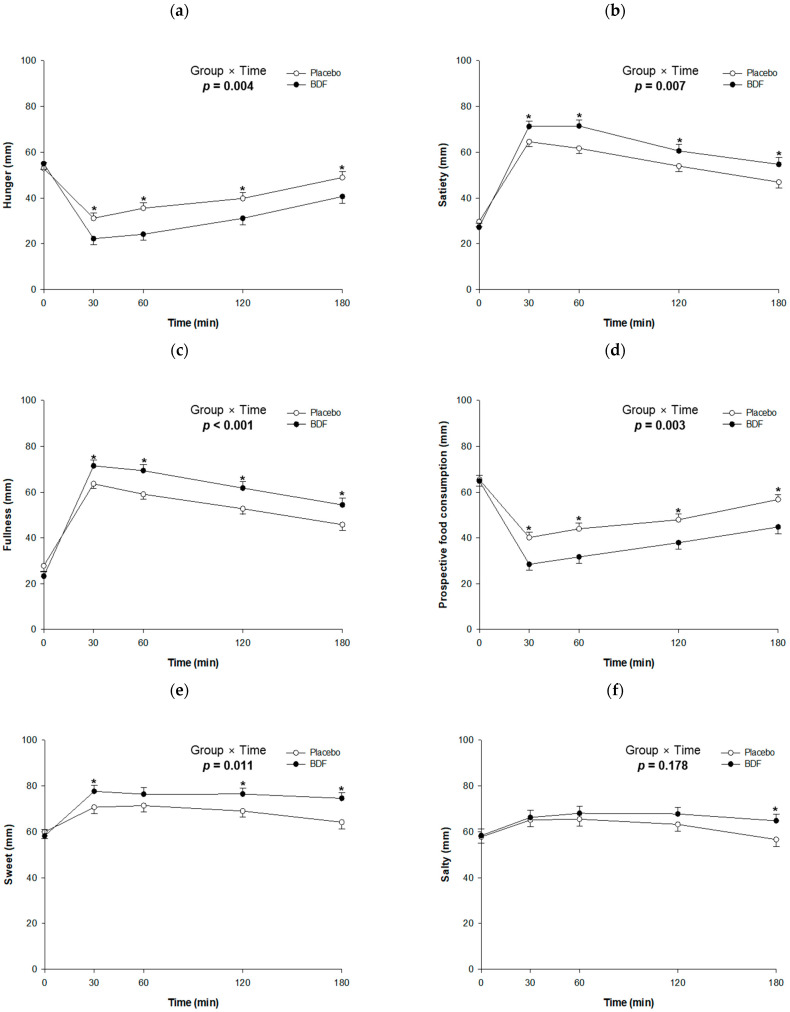

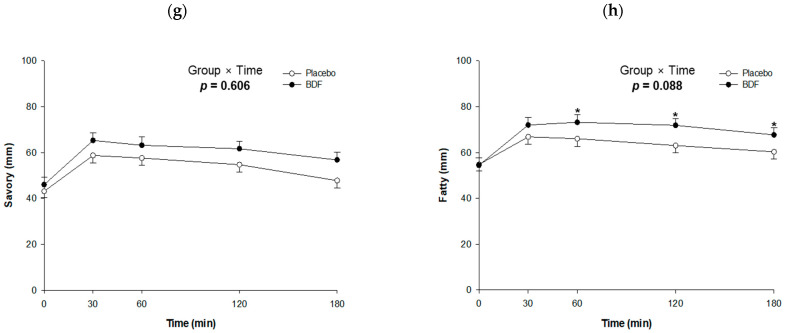
Effects of BDF on appetite parameters. (**a**) Hunger, (**b**) Satiety, (**c**) Fullness, (**d**) Prospective food consumption, (**e**) Sweet, (**f**) Salty, (**g**) Savory, (**h**) Fatty. Appetite measurements using the visual analog scale (VAS) showed comparable responses between the BDF and placebo groups. BDF, barley dietary fiber. Each line represents mean ± SE. Linear mixed-effect model was used to analyze the difference between the groups and the effects of group × time. * *p* value < 0.05.

Appetite measurements using VAS revealed similar responses between the two groups following the consumption of placebo or BDF with glucose loading. Notably, hunger levels in the BDF group were significantly lower than that in the placebo group, with reductions of −13.4 (*p* < 0.001) and −10.7 (*p* = 0.005) 30 and 60 min after consumption, respectively. In addition, prospective food consumption assessments indicated lower expectations for food intake in the BDF group, registering a decrease of −11.7 (*p* = 0.001) at 60 min. Conversely, satiety and fullness scores were higher in the BDF group, showing increases of 12.3 (*p* = 0.001) and 14.8 (*p* < 0.001), respectively, compared with the placebo group after 60 min, suggesting greater satisfaction after dietary intake. The sensory evaluations for sweet, salty, savory, and fatty flavors showed trends of high initial ratings that subsequently decreased over time, with no significant differences observed between the groups (Figure 4a–f).

**Figure 5:**
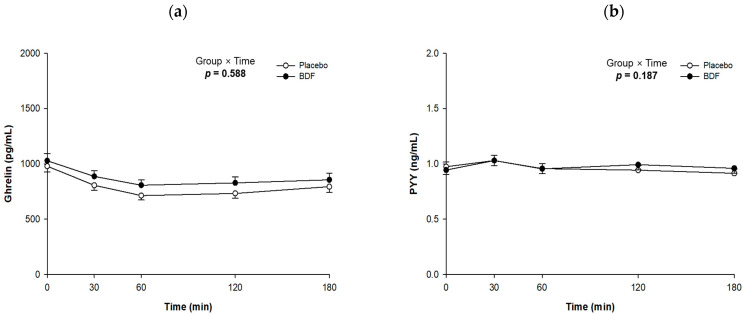
Effects of BDF on appetite hormones (**a**) ghrelin and (**b**) PYY Levels. BDF, barley dietary fiber. Each line represents mean ± SE. Linear mixed-effect model was used to analyze the difference between the groups and the effects of group × time.

Ghrelin levels were highest in both groups immediately before food intake, but they gradually decreased until 60 min post-consumption, after which they plateaued (Figure 5a). The BDF group showed a ghrelin concentration of 807.5 ± 48.7 pg/mL, while the placebo group had a level of 713.8 ± 40.4 pg/mL 60 min after glucose loading. The BDF group exhibited higher ghrelin levels, reaching −49.5 pg/mL; however, there was no significant difference between the groups.

PYY levels increased similarly in both groups 30 min after intake, coinciding with peak blood glucose and insulin levels (Figure 5b). However, PYY levels decreased after 60 min, reaching their lowest values after 180 min. Although PYY acts as an appetite-regulating hormone, no significant differences were found between the groups.

## 4. Discussion

This study was conducted to evaluate the effects and safety of barley dietary fiber (BDF) consumption on postprandial blood glucose, insulin, and appetite levels in healthy adults with fasting blood glucose levels below 126 mg/dL. Specifically, we investigated the effects of consuming Saechalssalbori (*Hordeum vulgare* L.), a barley variety with higher soluble dietary fiber content (9.5%), including β-glucan, and higher protein content (10.8%) compared with other glutinous or non-glutinous barley varieties [23]. In this study, the total dietary fiber content of Saechalssalbori was measured at an average of 16.44 g, with 5.55 g of soluble fiber, which is higher than the soluble dietary fiber content of polished barley (3.50–4.60 g) reported in the 2022 Korea Ministry of Food and Drug Safety Nutrition Database (MFDS, 2024). This variation can be attributed to differences in barley varieties, climate conditions, and cultivation regions.

The results showed that BDF consumption significantly delayed acute postprandial blood glucose increases compared with placebo consumption while suppressing the excessive secretion of endogenous insulin and slightly increasing glucagon and triglycerides. Additionally, we observed that ghrelin, the hunger-stimulating hormone, decreased, while PYY, which induces satiety, slightly increased. These findings align with previous studies that reported a low glycemic response (GI range of 21–40) following barley consumption [27] and the suppression of postprandial glucose levels by β-glucan from barley [12]. Furthermore, these results support existing research that dietary fiber, particularly β-glucan, can help mitigate acute postprandial blood glucose spikes [28,29].

The high water absorption and spreadability of the glutinous starch structure in Saechalssalbori, along with its ability to form a high-viscosity gel, likely contribute to its delayed gastric emptying and digestion in the intestines [22,30]. Additionally, the gel-like carbohydrates formed by the high-viscosity β-glucan can inhibit α-glucosidase in the small intestine, thereby slowing down digestion and delaying glucose absorption across the intestinal surface [9]. This mechanism helps explain the reduction in postprandial blood glucose and insulin secretion observed in this study [31,32].

Another hypothesis involves the fermentation of indigestible carbohydrates by gut microbiota, which produces short-chain fatty acids (SCFAs) like acetate, propionate, and butyrate. These SCFAs are known to improve glucose homeostasis and reduce appetite and body weight in rodents and humans [12]. This aligns with the findings of this study, which demonstrated that BDF consumption led to a significant reduction in postprandial blood glucose and insulin secretion compared with rice flour consumption.

The glucagon levels in the BDF group were slightly higher than in the placebo group, and the area under the curve (AUC) for glucagon was also greater in the BDF group, though the difference was not statistically significant (*p* = 0.001). This trend is consistent with previous findings that dietary fiber-rich meals, compared with meals consisting solely of white rice, can increase GLP-1 levels, suppress postprandial hyperglycemia, and reduce the excessive secretion of endogenous insulin [33]. Furthermore, our results show that barley flour intake, compared with placebo, may promote greater glucagon secretion, helping regulate blood glucose and insulin as a counter-regulatory mechanism [34].

In terms of triglyceride levels, the placebo group showed a reduction immediately after consumption, whereas the BDF group experienced a gradual increase. The AUC for triglycerides was also higher in the BDF group, showing a significant difference between the two groups. These results differ somewhat from those of Hong et al. [35], who found that β-glucan consumption reduced triglyceride levels in high-fat diets, suggesting that further investigation into the physicochemical properties of Saechalssalbori’s soluble fiber and its impact on lipid metabolism is needed.

The appetite measurement results indicated similar trends across both groups. However, hunger and prospective food consumption scores were significantly lower in the BDF group at 30–60 min post-consumption compared with the placebo group, while satiety and fullness scores were significantly higher in the BDF group. This supports the hypothesis that β-glucan-containing foods can induce greater satiety and suppress appetite [36,37]. The measurement of appetite fluctuations at 30 to 60 min intervals was chosen to capture both the immediate and sustained effects of barley BDF on hunger and satiety. Appetite regulation involves complex physiological processes, and short-term monitoring helps reveal how quickly BDF influences satiety signals post-consumption. The initial 30 min interval allows for the observation of early responses to BDF intake, which are likely influenced by rapid satiety signals from gastrointestinal and hormonal responses, including gut hormones such as GLP-1 and PYY [38]. Subsequent measurements at 60, 120, and 180 min provide insights into the persistence of BDF’s effects, as dietary fiber can delay gastric emptying, extending satiety over several hours [39]. These intervals were selected to understand BDF’s role in both immediate and longer-lasting appetite control, supporting the study’s objective to assess its potential in managing hunger and promoting satiety.

The correlation between VAS appetite ratings and physiological markers such as ghrelin, PYY, glucose, and insulin was noteworthy. Our study showed that ghrelin levels decreased after BDF consumption while PYY levels increased, which is consistent with findings from previous studies suggesting that ghrelin decreases and PYY increases are associated with satiety signals [40]. These findings provide valuable insights into the relationship between postprandial glucose, insulin, and appetite-related hormones such as ghrelin and PYY.

In this study, significant changes in subjective appetite scores VAS were observed following BDF consumption; however, these did not correspond with significant changes in the physiological markers ghrelin and PYY. This discrepancy may reflect the complex nature of appetite regulation, which involves not only circulating hormone levels but also neural pathways, sensory cues, and psychological influences. These additional factors may contribute to subjective sensations of hunger and satiety independently of ghrelin and PYY levels. Furthermore, individual variability in hormone sensitivity and postprandial metabolic responses may also play a role. Some individuals may experience satiety at lower hormone concentrations, while others may require higher levels to perceive similar effects. Additionally, BDF’s effects on satiety may involve the production of SCFAs and the release of other hormones, such as GLP-1, which were not measured in this study but may influence appetite independently of ghrelin and PYY.

In addition to the effects of β-glucan, BDF also contains phytate (inositol hexaphosphate), a bioactive compound known for its potential health benefits, particularly in the context of type 2 diabetes. Phytate has garnered attention for its role in reducing the formation of advanced glycation end-products (AGEs), which are associated with diabetic complications and oxidative stress [41]. Phytate has been shown to inhibit the glycation process by chelating metal ions that catalyze glycation reactions and by scavenging reactive oxygen species (ROS), thereby reducing oxidative stress [42]. This inhibition of AGE formation may help mitigate some of the long-term complications associated with diabetes, making phytate a valuable component of BDF for diabetic populations. Moreover, phytate has been reported to improve insulin sensitivity and regulate postprandial glucose levels. In studies on phytate-rich diets, reductions in blood glucose and improvements in insulin response were observed, likely due to phytate’s effects on slowing carbohydrate digestion and glucose absorption [43]. This delayed glucose absorption reduces postprandial spikes, which may be particularly beneficial for individuals managing blood glucose levels in diabetes.

However, there are several limitations of this study. First, the study was conducted with a relatively small sample size of 67 participants, primarily younger adults with an average age of 35.3 ± 1.1 years. This concentration of a younger age range may restrict the generalizability of the findings to other age groups, particularly older individuals or those with metabolic conditions such as type 2 diabetes. Additionally, there was a noticeable gender imbalance, with 55 male and 15 female participants. This skewed distribution could impact the applicability of the findings to a more gender-balanced population or to women specifically, who may exhibit different physiological responses.

Additionally, although the barley dietary fiber and rice flour placebo products were formulated to ensure consistency in dosing and delivery, there were small compositional differences beyond fiber content. Specifically, the barley product had slightly higher energy and fat content and a lower sugar content compared with the placebo. These differences may have had some influence on the outcomes, meaning that not all observed effects can be attributed solely to the fiber content of the barley product. Future studies could address this limitation by matching the macronutrient composition more closely between the test and placebo products to isolate the effects of dietary fiber more effectively.

Another limitation involves the reliance on self-reported appetite measures. Although the VAS scores provide useful insights into subjective appetite perceptions, these are self-reported measures and are therefore subject to individual biases. Future research could benefit from incorporating objective appetite measures to validate and support these findings. Furthermore, the study focused on measuring ghrelin and PYY as the primary hormones associated with appetite regulation, while other hormones, such as GLP-1 and GIP, were not included. GLP-1 and GIP play significant roles in both appetite regulation and glucose metabolism [43,44], and their inclusion in future studies could offer a more comprehensive understanding of the effects of BDF on appetite and metabolism.

Lastly, the relatively short duration of this study limits conclusions regarding the potential long-term metabolic benefits of BDF consumption. Studies with extended durations are needed to determine whether sustained intake of BDF could lead to improvements in long-term glycemic control, weight management, and overall metabolic health. These limitations highlight the need for cautious interpretation of the results and suggest directions for future research to build on these initial findings.

In conclusion, this study confirms the significant postprandial glucose-lowering effects and reduced insulin secretion following barley dietary fiber consumption, as well as its ability to increase glucagon levels and reduce hunger. These findings highlight the potential of barley dietary fiber as an effective dietary intervention for preventing and managing metabolic conditions such as diabetes by improving postprandial glucose control. Further studies are necessary to explore the long-term effects of consistent barley dietary fiber intake on glycemic variability and metabolic health.

## 5. Conclusions

This study demonstrates that the consumption of barley dietary fiber (BDF), particularly from Saechalssalbori, significantly lowers postprandial blood glucose and insulin levels while increasing glucagon secretion compared with rice flour. Additionally, BDF intake was shown to reduce hunger and increase satiety, potentially through the modulation of appetite-related hormones such as ghrelin and PYY. These findings suggest that barley dietary fiber, rich in β-glucan, may play a valuable role in managing postprandial glycemic responses and improving metabolic health. Given its positive effects on glucose metabolism and appetite control, BDF presents a promising dietary intervention for the prevention and management of diabetes, obesity, and related metabolic disorders.

## Figures and Tables

**Table 1:** Baseline characteristics of the 70 participants ^1^.

Variables	Value
Age (yr)	35.3 ± 1.1
Gender (male/female)	55/15
Menstruation (Y/N/NA)	15/0/55
RFS	17.3 ± 1.0
Body weight (kg)	72.4 ± 1.5
BMI (kg/m^2^)	24.4 ± 0.4
Waist circumference (cm)	79.9 ± 1.1
Alcohol drinker (Y/N)	44/26
Alcohol amount (SD/wk)	3.2 ± 0.5
Smoker (Y/N)	27/43
Smoking amount (cigarette/d)	2.8 ± 0.5
Physical activity (MET-min/wk)	2475 ± 328
Fasting glucose (mg/dL)	95.7 ± 0.9

^1^ Mean ± SE (all such values). BMI, body mass index; NA, not applicable; RFS, recommended food score; SD, standard drink.

**Table 2:** Postprandial blood glucose and insulin levels ^1^.

Variables	Placebo	BDF	Estimate ^2^	*p*-Value ^2^	*p*-Value ^3^
**Blood glucose (mg/dL)**					
0 min	93.4 ± 0.9	92.9 ± 1.0			
30 min	148.6 ± 2.3	122.2 ± 2.2	−26.0	<0.001	
60 min	137.9 ± 3.3	118.2 ± 2.5	−19.2	<0.001	
120 min	121.7 ± 2.5	111.8 ± 1.8	−9.4	0.004	
180 min	108.2 ± 1.8	104.7 ± 1.4	−3.0	0.342	<0.001
**AUC (mg/dL × min)**					
0–30	3629 ± 42	3226 ± 36	−398	<0.001	
30–60	4297 ± 78	3606 ± 66	−684	<0.001	
60–120	7785 ± 159	6902 ± 118	−869	<0.001	
120–180	6896 ± 112	6496 ± 84	−387	0.001	
0–30	3629 ± 42	3226 ± 36	−398	<0.001	
0–60	7926 ± 115	6832 ± 98	−1083	<0.001	
0–120	15,712 ± 264	13,734 ± 207	−1952	<0.001	
0–180	22,607 ± 357	20,230 ± 276	−2338	<0.001	
**C_max_ (mg/dL)**	152.3 ± 2.4	127.3 ± 2.2	−24.7	<0.001	
**T_max_ (min)**	41.0 ± 2.3	57.8 ± 4.8	17.0	0.002	
**Insulin (** **µU/mL)**					
0 min	7.4 0.7	7.3 ± 0.8			
30 min	70.9 ± 4.3	47.8 ± 3.6	−23.0	<0.001	
60 min	59.3 ± 3.9	39.0 ± 3.1	−20.3	<0.001	
120 min	52.7 ± 4.7	34.6 ± 2.5	−18.0	<0.001	
180 min	35.7 ± 2.7	28.2 ± 1.9	−7.5	0.095	<0.001
**AUC (** **µU/mL × min)**					
0–30	1175 ± 72	826 ± 59	−355	<0.001	
30–60	1954 ± 115	1302 ± 94	−670	<0.001	
60–120	3360 ± 234	2206 ± 158	−1186	<0.001	
120–180	2651 ± 208	1882 ± 123	−787	<0.001	
0–30	1175 ± 72	826 ± 59	−355	<0.001	
0–60	3129 ± 184	2128 ± 152	−1025	<0.001	
0–120	6489 ± 392	4334 ± 300	−2217	<0.001	
0–180	9140 ± 572	6216 ± 411	−3007	<0.001	
**C_max_ (** **µU/mL)**	79.8 ± 4.9	52.0 ± 3.5	−28.3	<0.001	
**T_max_ (min)**	53.8 ± 4.2	59.1 ± 5.3	5.5	0.395	

^1^ Mean ± SE (all such values). BDF, barley dietary fiber; AUC, area under the curve; C_max_, maximum concentration; T_max_, time to reach maximum concentration. ^2^ Linear mixed-effect model was used to analyze the difference between the groups. ^3^ Linear mixed-effect model was used to analyze the effects of group × time.

## Data Availability

The original contributions presented in the study are included in the article; further inquiries can be directed to the corresponding author.
